# Patients with appendicitis during COVID-19 pandemic: a retrospective cohort study

**DOI:** 10.1097/MS9.0000000000000618

**Published:** 2023-04-11

**Authors:** Elizabeth Ricard, Alexandre Marceau, Gabrielle Larouche, Heidi Dorval, François-Charles Malo

**Affiliations:** a Extern in Medicine; b Family Medicine Resident, Université Laval, Avenue De La Médecine, Québec; c General Surgery Resident; d Department of Surgery, Université De Sherbrooke, Sherbrooke, Canada

**Keywords:** appendectomy, appendicitis, complications, length of stay, postoperative

## Abstract

The Coronavirus Disease 19 (COVID-19) pandemic greatly affected the Quebec healthcare system from spring 2020 onward; the consultation delays that were generated may have delayed the management of urgent intra-abdominal pathologies. Our objective was to evaluate the impact of the pandemic on the length of stay and complications within 30 days of treatment of patients consulting for acute appendicitis (AA) at the *Centres intégrés universitaires de santé et de services sociaux* (CIUSSS) *de l’Estrie-Centre hospitalier universitaire de Sherbrooke* (Estrie-CHUS), Quebec, Canada.

**Methods:**

The authors conducted a single-center retrospective cohort study on the charts of all patients diagnosed with AA at the CIUSSS de l’Estrie-CHUS between March 13 and June 22, 2019 (control group) and between March 13 and June 22, 2020 (pandemic group). This corresponds to the first wave of COVID-19 in Quebec. Patients included were those with a radiologically confirmed diagnosis of AA. There was no exclusion criteria. Outcomes assessed were length of hospital stay and 30-day complications.

**Results:**

The authors analyzed the charts of 209 patients with AA (117 patients in the control group and 92 patients in the pandemic group). No statistically significant difference was observed for the length of stay or the complications between the groups. The only significant difference was the presence of hemodynamic instability on admission (22.2 vs. 41.3%, *P*=0.004) as well as a trend that did not reach statistical significance regarding the proportions of reoperation before 30 days (0.9 vs. 5.4%, *P*=0.060).

**Conclusion:**

In conclusion, the pandemic did not affect the length of stay of AA managed at the CIUSSS de l’Estrie-CHUS. It is not possible to conclude whether the first wave of the pandemic influenced complications related to AA.

## Background

Highlights

- We are the first Canadian study to focus on acute appendicitis (AA) management during Coronavirus Disease 19.
- The Coronavirus Disease 19 pandemic did not affect the length of stay of AA.
- Post-treatment complications of AA during the pandemic were reported.
- Each patient’s chart was carefully analyzed; administrative data was not used.

### COVID-19 in Quebec

In December 2019, the novel SARS-CoV-2 virus was first identified in Wuhan, China. Coronavirus Disease 19 (COVID-19) quickly took on global significance before being declared a pandemic by the WHO on March 11, 2020^1^. In an effort to curb the spread of the virus, Quebec’s public health authorities initially imposed measures that consisted of restricting travel between certain regions, closing daycare centers and schools, enforcing physical distancing measures, banning all gatherings, closing all services deemed nonessential, and interdiction of nonessential visits to long-term care centers and hospitals^2^. In Quebec, as elsewhere in the world, the virus has also led to a significant reorganization of the health system, and a decrease in emergency room consultations has been observed^3^,^4^.

### Description and management of acute appendicitis

Acute appendicitis (AA) is one of the most common causes of acute intra-abdominal pain worldwide. In Canada, its annual incidence varies between 80 and 111 cases per 100 000 people^5^. The main symptoms of AA are pain in the right lower quadrant, abdominal guarding, and periumbilical pain radiating to the right lower quadrant^6^. Laparoscopic appendectomy remains the treatment of choice, however, conservative treatment based on the administration of antibiotics (ATB) is now considered an interesting alternative for selected patients with uncomplicated appendicitis^7^. A complicated appendicitis is defined by the presence of a perforation or abscess on computed tomography or ultrasound; in the United States, this accounts for about 15–20% of AA cases^8^.

### Acute appendicitis and COVID-19

When properly managed, AA has low mortality and morbidity rates. However, it is recognized that these rates increase when there is a delay of treatment^9^,^10^. In the past months, many studies have looked at the impact of the pandemic on different aspects of AA, including its presentation, management, and associated complications. Most of these studies make the comparison between a group of patients suffering from AA during the COVID-19 pandemic and another group of patients who suffered the same pathology in previous years. The findings reported in the literature differ widely.

### Negative impacts of COVID-19 on patients with acute appendicitis

In the study by Burgard *et al.*, 65 cases of appendicitis treated during the pandemic were compared to 241 cases of AA treated in previous years. It has been shown that the rate of complicated appendicitis was higher during the pandemic period (52 vs. 20%, *P*<0.001). In addition, other significant differences were reported between the COVID-19 group and the control group: duration of symptoms greater than 48 h (61 vs. 26%, *P*<0.001), longer intervention time (77 vs. 61 min, *P*=0.002), length of stay longer than two days (63 vs. 32%, *P*<0.001), and duration of ATB treatment longer than three days (36 vs. 24%, *P*=0.001)^3^. The systematic review of 54 articles by Grossi *et al.* has similar results: the incidence of complicated appendicitis was increased in all age groups having received this diagnosis during the pandemic^11^. The same conclusions were also drawn in the study by Orthopoulos *et al*. Patients who underwent surgery for AA in 2020 and in 2019 were compared and a 45.5% decrease in uncomplicated appendicitis cases was observed between the pandemic and prepandemic groups while 21.1 and 29% increases were reported for perforated appendicitis and gangrenous appendicitis, respectively^12^.

### Positive or neutral impacts of COVID-19 on patients with acute appendicitis

However, other studies have come to the opposite conclusions. The study by Neufeld *et al.* concludes to a decrease in the incidence of uncomplicated appendicitis [RR=0.65, 95% CI (0.47–0.91)] without an increase in cases of complicated appendicitis [RR=0.89, 95% CI (0.52–1.52)]. In addition, the length of hospital stay was shorter by about half a day in the COVID group [RR=0.73, 95% CI (0.60–0.88)]^13^. Finally, the study by Köhler *et al.* 14 demonstrated no change between groups in terms of complicated appendicitis, post-treatment complication rates, or need for reoperation.

### Review of another Canadian study

These studies were conducted in many countries, but only one was done in Canada: the study of Gomez *et al.*, which was conducted in Ontario. Their main objective was to assess the rate of visits to the emergency room before and during the beginning of the pandemic compared to the rate of visits in 2019; their study is based on health administrative data only. Patients presenting with appendicitis, cholecystitis, ectopic pregnancy, or miscarriage were included. Concerning AA specifically, the study by Gomez *et al.* reported no significant differences between the groups, except for the incidence of AA, which was lower in the COVID group. The authors noted no difference between the groups in management, complications, and mortality^15^.

### Pertinence of our study

Our study stands out because it is the only one in Canada to be conducted on a cohort of patients whose focus is on the Quebec reality of different clinical aspects of AA in times of COVID-19. In addition, the in-depth analysis of the charts of each patient of our sample, rather than the use of health administrative data, will allow us to obtain results that are both reliable and detailed. We are also among the first studies to comprehensively report on post-treatment complications of AA during the pandemic, which is a major outcome for the patients.

### Objectives and hypothesis

The main and secondary objectives of our study are, respectively, to assess the impact of the pandemic on the length of hospital stay and complications within 30 days of surgical or medical treatment of patients consulting for AA at the *Centres intégrés universitaires de santé et de services sociaux* (CIUSSS) *de l’Estrie-Centre hospitalier universitaire de Sherbrooke* (Estrie-CHUS). Our hypothesis is that the presentation delays caused by the pandemic resulted in an increase in the severity of AA and, therefore, a longer hospital stay and more short-term complications. If our hypothesis turns out to be correct, the public health measures imposed on the population of Quebec in the spring of 2020 could therefore have had a harmful effect on patients with an acute health problem such as appendicitis.

## Methods

### Study design

We conducted a single-center retrospective cohort study on the patient’s charts of the CIUSSS de l’Estrie-CHUS, a teaching hospital. Patients included were those with a radiologically confirmed diagnosis of AA at the Hôtel-Dieu de Sherbrooke or Fleurimont Hospital; these hospitals are part of the CIUSSS de l’Estrie-CHUS. There was no exclusion criteria. We analyzed the files of all patients who presented with a diagnosis of AA during two time periods: a prepandemic period (control group) including all patients with AA between March 13 and June 22, 2019 and a pandemic period (study group) consisting of patients with the same diagnosis between March 13 and June 22, 2020. These time periods were chronologically identical to eliminate seasonal variability.

This period includes the first wave of COVID-19 because we wanted to assess the impact of this first wave only on patients consulting for appendicitis. As discussed in the Background section, Quebec’s public health authorities imposed measures during the first wave that consisted of restricting travel between certain regions, closing daycare centers and schools, enforcing physical distancing measures, banning all gatherings, closing all services deemed nonessential, and interdiction of visits to long-term care centers and hospitals^2^.

Because this study involved only anonymized data, patient confidentiality was ensured, and therefore consent was not required. The data was stored in a password-secured file on the principal investigator’s computer. The authors declare no conflict of interest.

### Variables measured

The variables measured to objectify the patients’ condition at admission were the time from symptom onset to consultation and the time from primary to surgical consultation, temperature, C-reactive protein, white blood cell count, hemodynamic instability, and peritonism. These variables were found in the initial consultation in the general surgery notes, or the emergency triage sheets.

With respect to radiological characteristics, the variables assessed were the type of imaging (computed tomography, ultrasound, or MRI), the presence of an abscess and its size if present, the diameter of the appendix, and the presence of a perforation, an appendicolith, or free fluid. These variables were found in the initial consultation in the general surgery notes, or in the radiology reports.

Management variables were the type of treatment (conservative or surgical), the type of surgery and its duration, the duration of intravenous ATB and the total duration of ATB in general, and the length of hospital stay. These variables were found in the patients’ summary sheets and the operating protocols.

The pathology of the appendix was also analyzed and categorized in AA, neoplasia, or appendix without inflammation. These variables were found in the operating protocols and the pathology reports.

Regarding complications within 30 days of treatment, the variables measured were the presence of a collection or ileus, wound infection, pulmonary, cardiac, or thromboembolic complications, reoperation, rehospitalization, admission to intensive care, and death. These variables were found in the summary sheets, the imaging reports, the operating protocols, or the visits within 30 days of the initial surgical consultations.

The data were extracted by a research assistant after approval by the ethics committee (*Comité d’éthique de la recherche du CIUSSS de l’Estrie-CHUS*), according to a standardized data extraction guide. The approval number of our study is 2021-3843. The work has been reported in line with the Strengthening the Reporting of cohort, cross-sectional and case-control studies in Surgery (STROCSS) 2021 Criteria^16^. Our registration’s unique identifying number is researchregistry8741^17^.

### Statistical analysis

Continuous data are presented as means with SD, whereas dichotomous data are presented as numbers and percentages. Continuous data were compared between the two groups using the Student *t*-test or the Mann–Whitney test depending on whether they were normally distributed or not. Dichotomous categorical data were compared with an *χ* ^2^ test or Fisher’s exact test. Statistical significance was set at *P* less than 0.05.

### Sample size calculation for primary outcome

Assuming a mean length of hospitalization of 2 days (before COVID), a SD of 2.25, an alpha (type 1 error) of 0.05, a power of 0.80, and a clinically significant difference of one day of hospitalization, 80 patients in each group is required to verify the primary outcome of the study.

## Results

We analyzed the files of 209 patients with AA. The control group consisted of 117 patients, while 92 patients formed the COVID group. Comparing the two groups, there were no statistically significant differences in sex, age, BMI, American Society of Anesthesiologists score, and some comorbidities (Table 1).

**Table 1:** Patients’ demographics

		2019 (control group) (*N*=117)	2020 (pandemic group) (*N*=92)	*P*
Sex	Female	49 (41.9)	46 (50)	0.265
	Male	68 (58.1)	46 (50)	
Age		35.26±19.718	34.50±20.609	0.788
BMI (kg/m^2^)		27.0±7.0	26.2±5.2	0.470
ASA score	1	64 (56.1)	51 (55.4)	0.169
	2	47 (41.2)	34 (37.0)	
	3	2 (1.8)	7 (7.6)	
	4	1 (0.9)	0 (0)	
Diabetes		5 (4.27)	2 (2.17)	0.469
COPD		1 (0.855)	5 (5.43)	0.089
Smoking		19 (16.24)	12 (13.19)	0.563
Coronary artery disease		3 (2.56)	4 (4.35)	0.702
Immunosuppression		2 (1.71)	1 (1.09)	1.000
Neurocognitive disorders		0 (0)	2 (2.17)	0.193

ASA, American Society of Anesthesiologists; COPD, chronic obstructive pulmonary disease.

Statistical significance was set at *P*<0.05.

The same was true for the consultation times, temperature, C-reactive protein measurement, leukocytes, and peritonism (Table 2). However, only 22.2% of patients in the control group presented with hemodynamic instability (heart rate > 100 bpm or systolic blood pressure <90 mmHg) compared to 41.3% of patients in the pandemic group (*P*=0.004).

**Table 2:** Presentation of patients at admission

	Year						
	2019 (control group)			2020 (pandemic group)			
	Mean	SD	*N* (%)	Mean	SD	*N* (%)	*P*
Symptoms – consultation delay (days)	2.19	2.31		2.47	2.70		0.421
First line consultation – surgery consultation delay (hours)	8.50	7.14		10.13	30.28		0.575
Temperature	37.4	0.8		37.5	0.9		0.401
CRP	61.23	77.70		63.92	78.07		0.817
Leukocytes	12.98	4.65		13.86	5.79		0.227
Hemodynamic instability			26 (22.2)			38 (41.3)	0.004
Peritonism			20 (17.1)			20 (21.7)	0.479

CRP, C-reactive protein; Hemodynamic instability = heart rate > 100 bpm or systolic blood pressure <90 mmHg.

Statistical significance was set at *P*<0.05.

On imaging, there was no statistically significant difference in the type of imaging used, the presence and size of an abscess, the diameter of the appendix, and the presence of an appendicolith or free fluid (Table 3).

**Table 3:** Radiological characteristics of appendix

		Year						
		2019 (control group)			2020 (pandemic group)			
		*N* (%)	Mean	SD	*N* (%)	Mean	SD	*P*
Type of imagery	None	1 (0.9)			0 (0)			0.649
	CT scan	42 (35.9)			35 (38.0)			
	Ultrasound	74 (63.2)			57 (62.0)			
	MRI	0 (0)			0 (0)			
Abscess		16 (13.8)			8 (8.7)			0.282
Appendix diameter			11.5	3.3		11.3	4.9	0.760
Abscess size			3.8	10.9		3.8	13.2	0.973
Appendicolith		40 (34.5)			30 (32.6)			0.883
Free fluid		54 (46.6)			41 (44.6)			0.781

CT, computed tomography.

Statistical significance was set at *P*<0.05.

There were no statistically significant differences in the management of AA (Table 4). Most patients in the control and COVID groups underwent laparoscopic appendectomy, 97.4 and 98.9%, respectively. The length of stay was similar between the groups (2±3 days, *P*=0.630).

**Table 4:** Management of acute appendicitis

		Year						
		2019 (control group)			2020 (pandemic group)			
		*N* (%)	Mean	SD	*N* (%)	Mean	SD	*P*
Type of management	ATB alone	1 (0.9)			2 (2.2)			0.673
	X-Ray drain alone	1 (0.9)			0 (0)			
	Surgical	115 (98.3)			90 (97.8)			
Type of surgery	Laparoscopic appendicectomy	112 (97.4)			89 (98.9)			0.303
	Laparotomy appendicectomy	2 (1.7)			0 (0)			
	Right hemi-colectomy or ileocecal resection	0 (0)			1 (1.1)			
	Draining + washing alone	1 (0.9)			0 (0)			
Duration of surgery (min)			49	20		52	25	0.325
Duration of intravenous ATB (days)			1.74	2.33		2.12	3.19	0.324
Total duration of ATB (days)			3.08	4.15		4.61	7.86	0.073
Length of stay (days)			2	3		2	3	0.630

ATB, antibiotics; min, minutes.

Statistical significance was set at *P*<0.05.

After pathology analysis, we observed that 98.3% of the patients in the control group had a confirmed AA, while 95.6% of the patients in the pandemic group did (*P*=0.318) (Table 5).

**Table 5:** Pathology of the appendix

		Year		
		*N* (%)		
		2019 (control group)	2020 (pandemic group)	*P*
Pathology	Acute appendicitis	113 (98.3)	87 (95.6)	0.318
	Neoplasia	1 (0.9)	2 (2.2)	
	Appendix without inflammation	1 (0.9)	2 (2.2)	

Statistical significance was set at *P*<0.05.

No statistically significant differences could be observed between the groups regarding post-treatment complications (Table 6). Regarding reoperation within 30 days, we observed a trend, but it did not reach statistical significance. Indeed, 0.9% of the patients in the control group were reoperated compared to 5.4% of the patients in the COVID group (*P*=0.060).

**Table 6:** Post-treatment complications of acute appendicitis

	Year		
	*N* (%)		
	2019 (control group)	2020 (pandemic group)	*P*
Collection	6 (5.1)	8 (8.7)	0.405
Ileus	5 (4.3)	6 (6.5)	0.541
Wound infection <30 days	1 (0.9)	3 (3.3)	0.322
Pulmonary complication <30 days	2 (1.7)	2 (2.2)	0.594
Cardiac complication <30 days	1 (0.9)	1 (1.1)	0.688
Thromboembolic complication <30 days	0 (0)	0 (0)	—
Reoperation <30 days	1 (0.9)	5 (5.4)	0.060
Rehospitalization <30 days	7 (6.0)	8 (8.7)	0.591
Admission to intensive care <30 days	3 (2.6)	3 (3.3)	0.540
Death <30 days	0 (0)	1 (1.1)	0.440

Statistical significance was set at *P*<0.05.

## Discussion

The objective of this study was to evaluate whether the COVID-19 pandemic had an impact on the length of hospital stay and post-treatment complications of patients presenting with AA to the CIUSSS de l’Estrie-CHUS. We are the first Canadian study to focus on this specific pathology and to analyze the charts of each patient of our large sample rather than using administrative data. We are also among the first to report in detail on post-treatment complications of AA during the pandemic, which is a major outcome for the patients.

We analyzed data from the period between March 13 and June 22, which corresponds to the first wave of COVID-19 in Quebec. We felt that studying a longer period of time would have added too much heterogeneity to the results, as the health measures, and restrictions were variable in subsequent waves. For example, during the summer of 2020, restaurants reopened, almost all sectors of economic activities recovered, and gatherings were slowly authorized. The mandatory wearing of a mask was also introduced in this period^2^. Moreover, the population’s vision and sense of danger regarding the pandemic were certainly very different from what was experienced in the first few weeks.

### Primary and secondary outcomes

In this study, we found no statistically significant difference between the length of stay of patients in the control and pandemic groups; this observation is consistent with the results of previous studies^18^–^20^. This may be explained by the fact that at the beginning of the pandemic, postoperative patients were encouraged to leave the hospital quickly to reduce their risk of contracting COVID-19. In addition, because the rate of complicated appendicitis in the COVID group was similar to the one in the control group, the care provided was routine, and therefore there was no need for longer hospitalization. In this study, we also did not observe statistically significant differences in post-treatment complications between the control and COVID groups; this is also the case in previous studies^14^,^15^.

The similarity between our pre- and per-COVID patient cohorts, particularly regarding the management, could explain these nonstatistically significant results in terms of post-treatment complications. Indeed, in Sherbrooke, the treatment of choice for AA remained laparoscopic appendectomy; it was not replaced by treatment with ATB. Early in the pandemic, the American College of Surgeons and the Royal College of Surgeons of England issued new guidelines suggesting treating uncomplicated appendicitis with antibiotic therapy rather than appendectomy^21^,^22^. Despite this, there are questions about a possible association between conservative treatment and post-treatment complications. Indeed, it is known that the failure rate of nonsurgical treatment varies between 5 and 35%^23^–^26^. Thus, the surgical approach that was favoured in Sherbrooke may have contributed to limiting the number of post-treatment complications, which would explain our nonstatistically significant results between our control and COVID groups.

### Other outcomes

We observed a significant number of patients presenting with hemodynamic instability during the pandemic. This could be explained by the fact that patients waited until they were more symptomatic before consulting, by fear of going to the hospital and being exposed to the virus. However, consultation delays were similar between the control and pandemic groups, but the mean consultation delay was slightly longer in the COVID group.

Finally, the rate of reoperation at less than 30 days was higher in the COVID group, but this was a trend and not a significant result. This result is consistent with El Nakeeb’s study, which also found an increased reoperation rate in this same group^27^. However, this study also found an increase in complications (abscess, peritonitis, or mass) in their COVID group, unlike ours, which justified their higher reoperation rate.

### Limitations

First of all, the retrospective nature of our study is a limitation itself. Indeed, some variables were sometimes absent from the patients’ files and could not be measured. Second, the sample size and the possibility of comparing with other studies that studied a longer period is a weakness of our study. However, it is also a strength because it limited the heterogeneity and allowed us to study a very precise moment of the pandemic where there were the most restrictions and especially a certain fear of the population in front of COVID-19’s beginnings.

In addition, there may have been a timing bias: since our two cohorts were not studied in the same year, there may have been changes in practice or administrative changes. To our knowledge, there were no such changes and the two periods are close enough chronologically to limit this bias. Moreover, the power of our study was frankly insufficient to detect a difference in post-treatment complications. However, this was only a secondary exploratory objective. Finally, our external validity was limited to the Quebec context, given the demographic characteristics and management of our patients.

## Conclusion

In conclusion, the pandemic did not affect the length of stay of AA managed at the CIUSSS de l’Estrie-CHUS. It is not possible to conclude whether the first wave of the pandemic influenced complications related to AA.

## Ethical approval

The data were extracted after approval by the ethics committee, according to a standardized data extraction guide.

## Consent

Because this study involved only anonymized data, patient confidentiality was ensured and therefore consent was not required.

## Sources of funding

No grant support has been attributed to this study.

## Author contribution

E.R. : analysis and interpretation, writing of article; A.M.: conception and design, data collection, analysis and interpretation; G.L.: conception and design, data collection, analysis and interpretation; H.D.: conception and design, data collection, analysis and interpretation; F.-C.M.: conception and design, data collection, analysis and interpretation.

## Conflicts of interest disclosure

The authors declare that they have no conflict of interest.

## Guarantor

Elizabeth Ricard. François-Charles Malo.

## Provenance and peer review

Not commissioned, externally peer reviewed.
